# Different Frequency Bands in Various Regions of the Brain Play Different Roles in the Onset and Wake-Sleep Stages of Infantile Spasms

**DOI:** 10.3389/fped.2022.878099

**Published:** 2022-05-12

**Authors:** Yan Dong, Ruijuan Xu, Yaodong Zhang, Yali Shi, Kaixian Du, Tianming Jia, Jun Wang, Fang Wang

**Affiliations:** ^1^Henan Key Laboratory of Child Brain Injury and Henan Pediatric Clinical Research Center, The Third Affiliated Hospital and Institute of Neuroscience, Zhengzhou, China; ^2^Henan Key Laboratory of Children's Genetics and Metabolic Diseases, Henan Neurodevelopment Engineering Research Center for Children, Children's Hospital Affiliated to Zhengzhou University, Zhengzhou, China; ^3^Department of Children's Rehabilitation, The Third Affiliated Hospital of Zhengzhou University, Zhengzhou, China; ^4^Department of Medical Record Management, The Third Affiliated Hospital of Zhengzhou University, Zhengzhou, China

**Keywords:** EEGs analysis, infantile spasms, local network, global network, functional brain networks

## Abstract

**Objective:**

The study aimed to identify the signatures of brain networks using electroencephalogram (EEG) in patients with infantile spasms (IS).

**Methods:**

Scalp EEGs of subjects with IS were prospectively collected in the first year of life (*n* = 8; age range 4–8 months; 3 males, 5 females). Ten minutes of ictal and interictal EEGs were clipped and filtered into different EEG frequency bands. The values of each pair of EEG channels were directly compared between ictal with interictal onsets and the sleep-wake phase to calculate IS brain network attributes: characteristic path length (CPL), node degree (ND), clustering coefficient (CC), and betweenness centrality (BC).

**Results:**

CPL, ND, and CC of the fast waves decreased while BC increased. CPL and BC of the slow waves decreased, while ND and CC increased during the IS ictal onset (*P* < 0.05). CPL of the alpha decreased, and BC increased during the waking time (*P* < 0.05).

**Conclusion:**

The transmission capability of the fast waves, the local connectivity, and the defense capability of the slow waves during the IS ictal onset were enhanced. The alpha band played the most important role in both the global and local networks during the waking time. These may represent the brain network signatures of IS.

## Introduction

Infantile spasms (IS), which have been associated with development arrest or regression, are increasingly recognized as a specific type of seizure characterized by hypsarrhythmia on electroencephalogram (EEG) (1). It is the largest single epilepsy subgroup affecting 13–45.5% of infants (2) with an annual incidence of 32.7/100 000 live births (3). In recent years, more and more studies have been conducted to discover that on scalp EEG, γ oscillations can better detect susceptibility to epilepsy than ripple and fast ripple oscillations in IS children (4). High-frequency oscillations (HFOs) and coupling between HFOs and slow-wave activity can trigger spasms (5). Davis and colleagues have suggested that overconnectivity may reflect progressive pathologic network synchronization culminating in generalized epilepsy spasm (ES) when the stage II or quiet sleep EEG was clipped (6). It is widely recognized that IS is characterized by brain network abnormalities (1, 7), but research on the modifications of the spasm onset remains in the exploratory stage. It is also difficult to differentiate between waking and sleeping states on scalp EEG with hypsarrhythmia (1). Therefore, accurately analyzing EEG signatures is important to diagnose this condition.

Brain connectivity datasets comprise networks of regions connected by functional associations or anatomical tracts (8–11). A growing body of research has suggested that IS is associated with brain networks (7, 12–15). Shrey et al. (7) have retrospectively identified ES patients and found that varied functional networks could be used as objective markers of treatment response. A recent study has demonstrated that multifocal interictal spikes and high-amplitude slow-wave activity within the hypsarrhythmia are associated with the activation of different neuronal networks (16). However, the detailed mechanism of hypsarrhythmia is unclear. Spikes cause a cortical activation pattern, and slow-wave activity produces a hypsarrhythmia-specific activation in the cortex and subcortical structures—such as the brainstem, thalamus, and putamen—but the changes in brain network attributes other than network connectivity are unknown (6). Furthermore, there has been no clear mechanism underlying the IS onset and differences between the waking and sleep phases, especially based on hypsarrhythmia on EEG.

We investigated abnormal neural connectivity in IS patients at the IS onset or upon waking and sleeping stages to determine whether the processes could be quantified with network measures of scalp EEG data. The results provide helpful insights into the mechanisms and characteristics of functional brain networks in IS.

## Methods

### Subjects

Eight patients (3 males and 5 females) with a diagnosis of IS were enrolled in the study when admitted to our hospital from October 2019 to August 2020. The diagnosis of IS was made according to criteria proposed by the International League Against Epilepsy (ILAE) (17). The inclusion criteria were (1) onset age of <12 months with typical clinical symptoms including sudden or transient ES with synchrony or asymmetry in the neck, trunk, and limbs; (2) interictal EEG showing hypsarrhythmia; (3) profound developmental retardation or regression; and (4) no prior treatment with hormone drugs. The exclusion criteria were (1) neurodegenerative diseases, (2) associations with other severe diseases (e.g., severe liver or kidney dysfunction or severe heart failure), (3) early treatment termination for various reasons (adverse reactions or personal reasons); (4) parents' request for withdrawal from clinical observation, or (5) incomplete clinical data.

**Figure 1:**
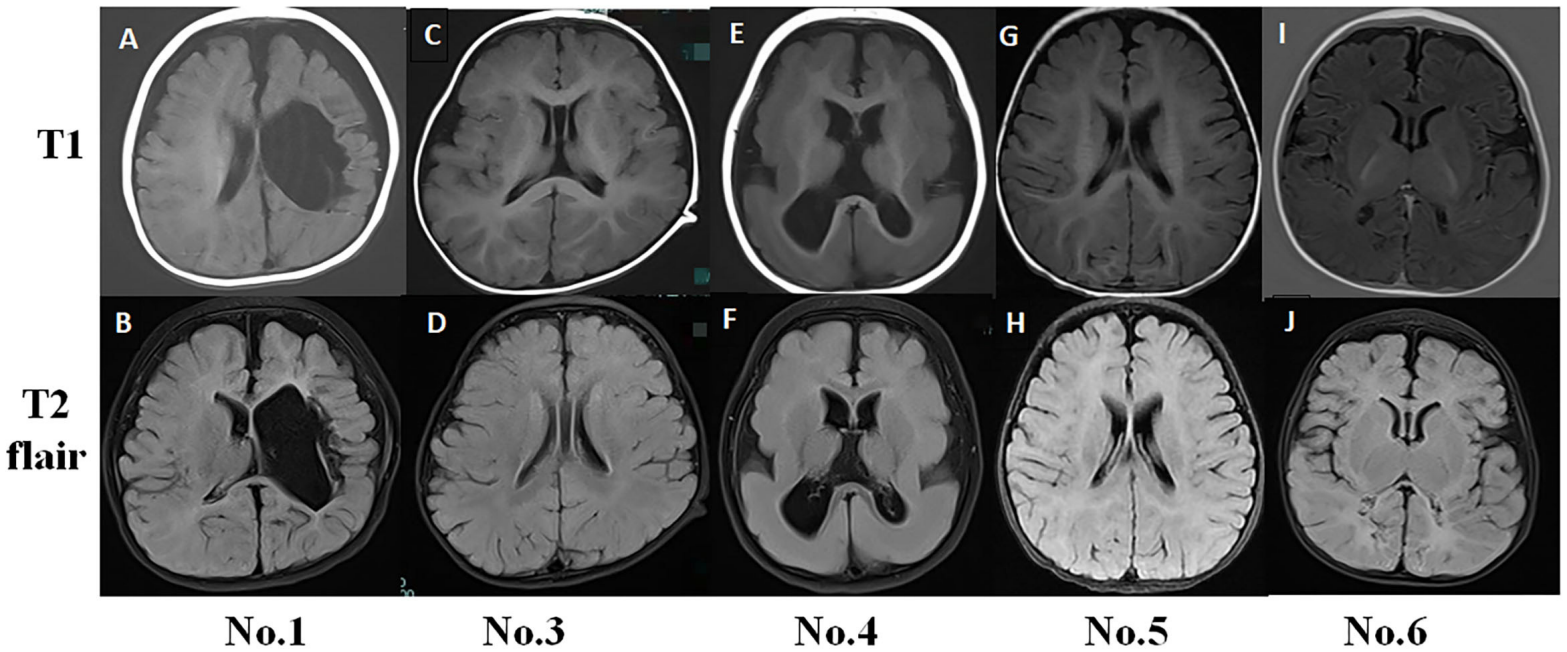
Typical magnetic resonance imaging (MRI) findings in infantile spasm (IS) patients. **(A,B)** No. 1 MRI (8-month-old) showed left hemisphere softening, left ventricle deformity, and cerebral peduncle atrophy. **(C,D)** No. 3 MRI (15-month-old) had normal findings. **(E,F)** No. 4 MRI (11-month-old) showed pachygyria. **(G,H)** No. 5 MRI (6-month-old) had normal findings. **(I,J)** No. 6 MRI (4-month-old) exhibited abnormal signals in the bilateral occipital and parietal lobes.

The seizure type was confirmed by video-EEG (VEEG). The burden of amplitudes and epileptiform discharges (BASED) score was used to evaluate IS-associated hypsarrhythmia on EEG (18, 19). All 8 patients had frequent ES (daily attacks). All patients were treated with antiseizure medication but had not previously been treated with adrenocorticotropic hormone (ACTH) or glucocorticoids. The patients' clinical data, neuropsychological and laboratory test results, magnetic resonance imaging (MRI) findings, and EEG reports were retrospectively collected and summarized. Further clinical details are demonstrated in Table 1, Figure 1.

**Table 1:** Subjects' demographics and clinical data.

	**Sex**	**AAO**	**AAST**	**ST**	**DS**	**ABT**	**EEG**		**MRI findings**	**Etiology**	**ROF**	**TOF**
							**Manifestation**	**BASED score**				
1	F	7	8	Spasm	PR	N	Left region hypsarrhythmia	4	Left hemisphere softening, left ventricle deformity, and cerebral peduncle atrophy	Structural	Seizure free	13
2	M	4	4	Spasm	PR	N	Central, parietal, and temporal hypsarrhythmia	4	Bilateral frontal, temporal, and occipital atrophy	Structural	Seizure	21
3	F	8	15	Spasm	PR	N	Hypsarrhythmia	5	N	Genetic	Seizure	14
4	M	6	6	Spasm, spasm-stiffness	PR	N	Discontinuous hypsarrhythmia	4	Pachygyria	Structural	Seizure free	11
5	F	5	6	Spasm	PR	N	Discontinuous hypsarrhythmia	4	N	Unknown	Seizure	15
6	F	6	7	Spasm	PR	N	Hypsarrhythmia	5	Abnormal signal in the bilateral, occipital, and parietal lobes	Structural	Seizure free	12
7	M	4	5	Spasm	PR	N	Hypsarrhythmia	5	Abnormal signal in the bilateral thalamus and pons; widening of the frontal sulcus	Structural	Seizure free	13
8	F	7	8	Spasm	PR	N	Discontinuous hypsarrhythmia	4	N	Unknown	Seizure free	17

*AAO, age at onset (months); AAST, age at the start of treatment (months); ABT, abnormal blood tests; BASED score, Burden of Amplitudes and Epileptiform Discharges score; DS, development state at start of observation period; EEG, electroencephalogram; MRI, magnetic resonance imaging; N, none; PR, psychomotor retardation; ROF, result of follow-up; ST, seizure type; TOF, time of follow-up (months)*.

### EEG Data Acquisition

Scalp VEEG (16/24 h) data were obtained with electrodes placed according to the 10–20 international system of electrode placement at a sampling rate of 500 Hz (Nihon-Kohden, Tokyo, Japan). The delta (0.3–3.5 Hz), theta (4–7.5 Hz), alpha (8–13 Hz), beta (14–30 Hz), and gamma waves (30–70 Hz) were analyzed by two board-certified clinical neurophysiologists. Discrepancies between the readers were resolved by a third board-certified clinical neurophysiologist.

Ten-minute ictal (including one or more ES) and interictal EEGs were clipped and filtered into canonical EEG frequency bands. The chosen EEGs were divided into 300 epochs (1 epoch per 2 s). Mutual information values for each pair of EEG channels were directly compared between the ictal and interictal phases, awake and sleep periods of characteristic path length (CPL), node degree (ND), clustering coefficient (CC), and betweenness centrality (BC).

### Data Preprocessing

An extensive introduction to graph theoretical measures can be found in a previous study (8). We performed the following steps for the original EEG. First, we conducted an average reference and then used the EEGLAB (https://sccn.ucsd.edu/eeglab/index.php) toolkit for bandpass filtering (0.5–45 Hz). Second, an independent component analysis was performed on the filtered signals. Then, we used the SASICA (https://eeglab.org/others/EEGLAB_Extensions.html) toolkit to remove artifact data to obtain the preprocessed EEG signal. Finally, we used the BCT toolkit (https://www.nitrc.org/projects/bct/) to obtain the CPL, ND, CC, and BC.

Briefly, CPL represents information transmission of the whole-brain network and denotes the average path length of all nodes in the network. It can be described as $L^{W}=\frac{1}{n}\sum_{i\in N}\frac{\sum_{j\in N,j\neq i}d_{ij}^{w}}{n-1}$, where *N* is the set of all nodes in the network; *n* is the number of nodes; *L* is the set of all links in the network; links (*i, j*) are associated with connection weights w_*ij*_ (0 ≤ w_*ij*_ ≤ 1 for all *i* and *j*); and d_*ij*_ is the length of the shortest path between *i* and *j*. *l*^*w*^ is the sum of all weights in the network, computed as *l*^*w*^ = ∑_*i, j*∈*N*_*w*_*ij*_. ND reflects the local connectivity of the node in the local network, being computed as $k_{i}^{w}=\sum_{j\in N}w_{ij}$, and $k_{i}^{w}$ is the weighted degree of *i*. CC measures the local information transmission capacity of the network and reflects the defensive ability of the local network to defend against random attacks. It can be described as $C^{w}=\frac{1}{n}\sum_{i\in N}\frac{2t_{i}^{w}}{k_{i\left(ki-1\right)}}$, where *C*^*w*^ is the weighted clustering coefficient and $t_{i}^{w}$ is the weighted geometric mean of triangles around *i*. BC, a measure of a node in the degree of centrality of the local network as a hub in the network and computed equivalently on weighted and directed networks, defines node centrality from the perspective of information flow (8, 20). It can be described as $C_{b}\left(i\right)=\underset{j<k}{\sum }\frac{g_{jk}\left(i\right)}{g_{jk}}$, where *g*_*jk*_ is the total number of shortest paths from nodes *j* and *k* and *g*_*jk*_(*i*) is the number of those paths that pass through the node *i* (12).

### Group Statistical Comparisons

**Figure 2:**
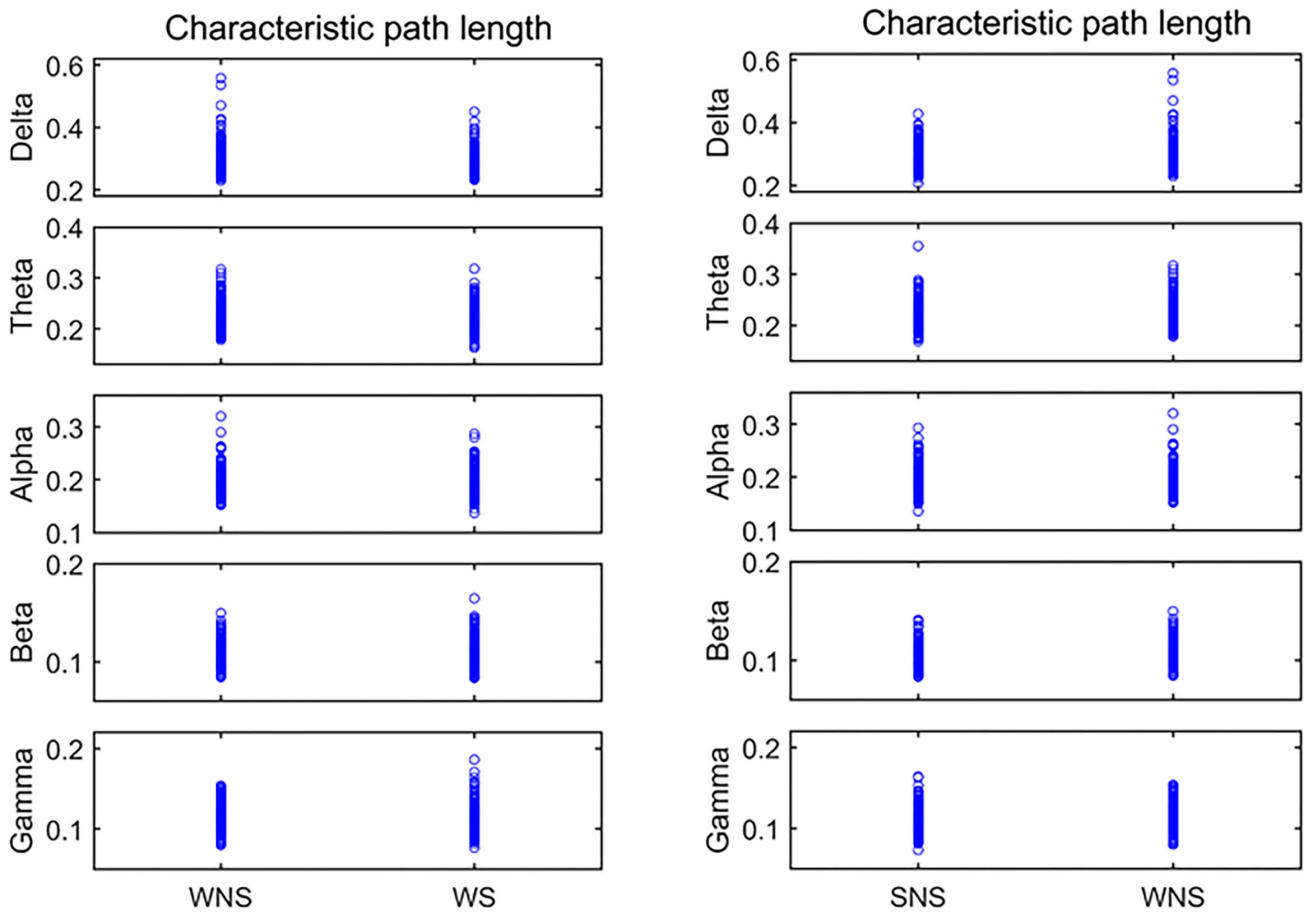
Characteristic path length (CPL) in different frequency bands. Two-sided non-parametric Wilcoxon rank-sum tests were taken. The delta, alpha, and beta frequencies became shorter when comparing the ictal and interictal phases, and the alpha band expanded when comparing the waking and sleep phases (*P* < 0.05). SNS, sleep period; WNS, awake period; WS, ictal period during waking.

**Figure 3:**
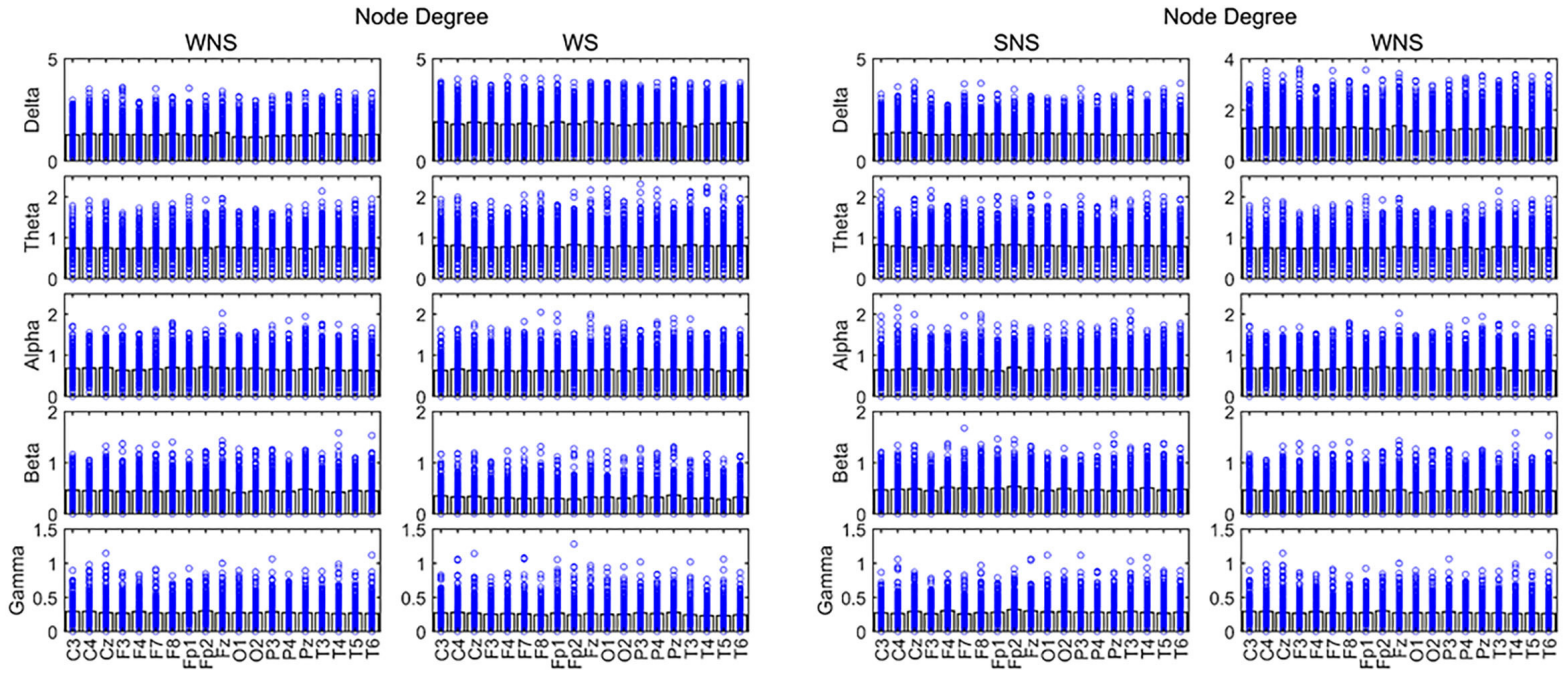
Node degree (ND) in different frequency bands. Two-sided non-parametric Wilcoxon rank-sum tests were taken. Connectivity capabilities of slow waves in the forehead increased in the ictal phase (*P* < 0.05). The alpha band was stronger, while slow waves and the beta band were weaker in local EEG connectivity (*P* < 0.05). SNS, sleep period; WNS, awake period; WS, ictal period during waking.

**Figure 4:**
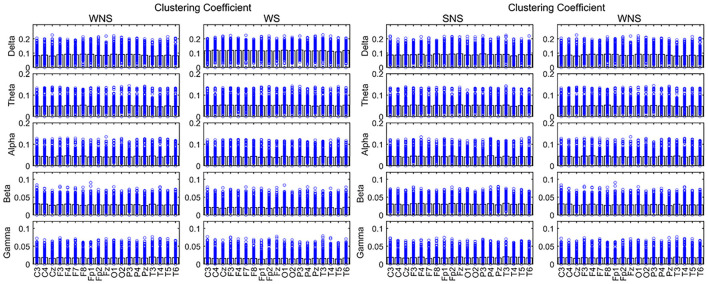
Clustering coefficient (CC) in different frequency bands. Two-sided nonparametric Wilcoxon rank-sum tests were taken. The defense capabilities of slow waves increased (*P* < 0.05). The slow waves and the beta band were weaker in local EEG defense capabilities, and the alpha band of Pz was stronger in local networks while patients were awake (*P* < 0.05). SNS, sleep period; WNS, awake period; WS, ictal period during waking.

**Figure 5:**
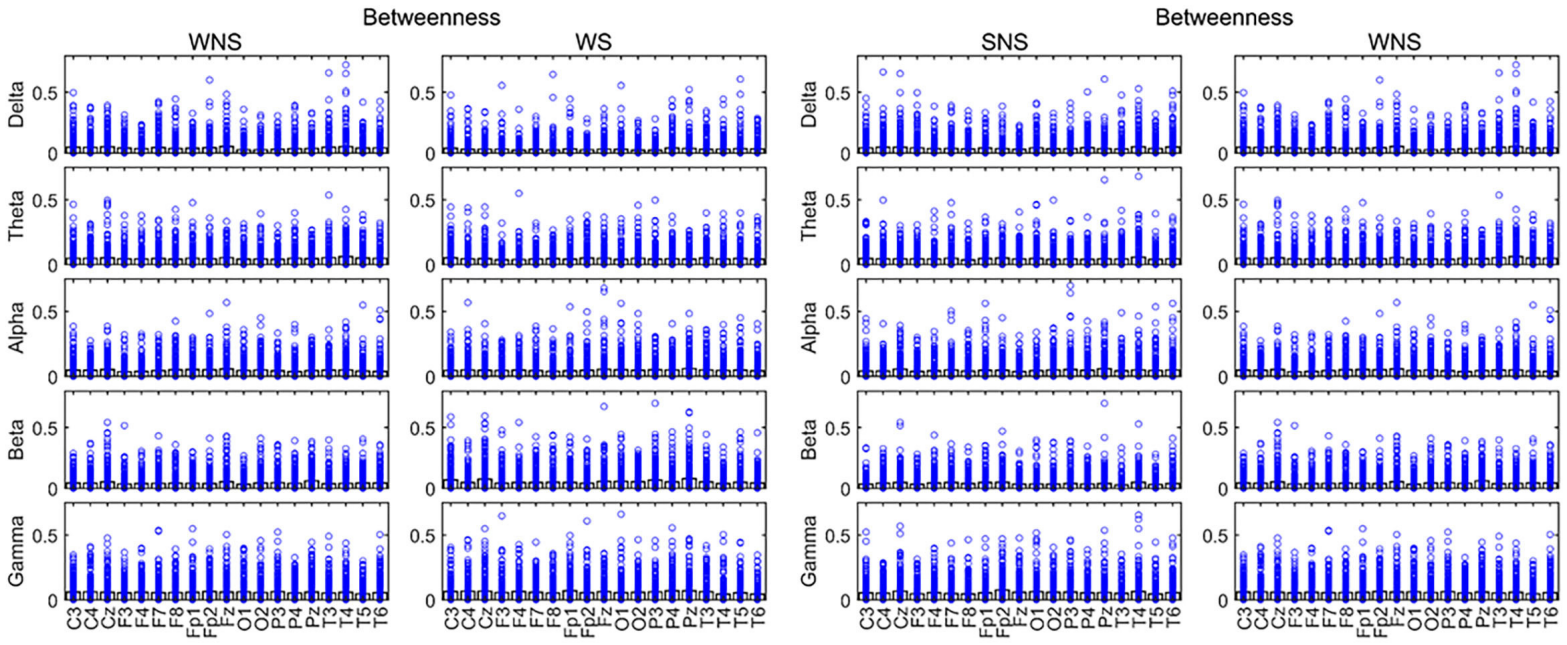
Betweenness centrality (BC) in different frequency bands. Fast waves played more important roles in the centrality of the local network, especially in the forehead in the IS ictal phase (*P* < 0.05). SNS, sleep period; WNS, awake period; WS, ictal period during waking.

All data and statistical analyses were performed using MATLAB software (the MathWorks Inc., Natick, MA, USA). Brain functional network attributes were compared with two-sided non-parametric Wilcoxon rank-sum tests (Supplementary Tables 2, 3, Figures 2–**5**). Statistical significance was set at *P* < 0.05. Brain networks based on EEG signals were compared across the ictal and interictal phases and the waking and sleep phases.

## Results

### Clinical Data

The clinical characteristics of IS patients are shown in Table 1. There were 3 males and 5 females. The age of onset of IS children was 4.42–7.34 (5.88 ± 1.46) months, while the age at the start of treatment was 6.50 (5.25–8.00) months. All 8 patients had psychomotor retardation. The EEGs of 3 patients showed hypsarrhythmia, while the other 5 patients' EEGs showed atypical hypsarrhythmia. The mean BASED score was 5.88 ±1.46. The etiology was structural in 5 patients (62.50%), unknown in 2 (25.00%), and genetic (Down syndrome) in 1 (12.50%). Five patients (62.50%) were controlled (Table 1) over more than 12-months of follow-up.

### Typical MRI Examinations

No. 1 MRI T1 and T2 flair examination showed left hemisphere softening, left ventricle deformity, and cerebral peduncle atrophy. The MRI findings for No. 3 and No. 5 were normal. No. 4 MRI showed pachygyria (Figure 1).

### CPL Differences Between the Groups

Comparison of the CPL values of the ictal and interictal phases showed that the delta, alpha, and beta frequencies became shorter, while the alpha band was shorter relative to CPL when comparing the waking and sleep phases (*P* < 0.05), as shown in Figure 2, Tables 2, 3.

**Table 2:** Characteristics of brain networks in different frequency bands of IS compared WS with WNS stage.

	**Delta**	**Theta**	**Alpha**	**Beta**	**Gamma**
CPL	↓^*^		↓^*^	↓^*^	
ND	FP1-Pz↑^*^	Fp2↑^*^, C3↑^*^, C4↑^*^	FP1↓^*^, FP2↓^*^, C3↓^*^, Cz↓^*^, F7↓^*^, F8↓^*^, O2↓^*^	FP1-Pz↓^*^	FP2↓^*^, F4↓^*^, F8↓^*^, T3↓^*^, T4↓^*^, T5↓^*^, O2↓^*^
CC	FP1-Pz↑^*^	T4↑^*^, T6↑^*^, Fz↑^*^, Cz↑^*^	F4↓^*^, Pz↓^*^	FP1-Pz↓^*^	FP1↓^*^, FP2↓^*^, C4↓^*^, P4↓^*^, T3↓^*^, T5↓^*^, O2↓^*^
BC	FP1↓^*^, FP2↓^*^, F3↓^*^, F4↓^*^, C4↓^*^, F7↓^*^, F8↓^*^, T3↓^*^, T4↓^*^, T6↓^*^, Fz↓^*^, Cz↓^*^	T4↓^*^	FP1↑^*^, F4↑^*^, P4↑^*^	C3↑^*^, P3↑^*^, Pz↑^*^, O1↑^*^	FP2↑^*^, P4↑^*^, Pz↑^*^, T4↑^*^

*Two-sided non-parametric Wilcoxon rank-sum tests were taken*.

* *Indicates statistical significance at P < 0.05*;

*↑ represents increasing or expanding, and*

*↓ represents decreasing or shortening*.

*BC, betweenness centrality; CC, clustering coefficient; CPL, characteristic path length; ND, node degree; WNS, awake period; WS, ictal period during waking*.

**Table 3:** Characteristics of brain networks in different frequency bands of IS between the WNS and SNS stages.

	**Delta**	**Theta**	**Alpha**	**Beta**	**Gamma**
CPL			↓^*^		
ND	P3↓^*^,O1↓^*^, O2↓^*^	FP1↓^*^,FP2↓^*^,F4↓^*^, C3↓^*^	FP1↑^*^,C4↑^*^,T6↓^*^	FP2↓^*^, F4↓^*^, F7↓^*^, F8↓^*^, T4↓^*^,T6↓^*^, O1↓^*^,O2↓^*^	C4↑^*^,Fz↓^*^
CC	FP2↓^*^, Fz↓^*^, Cz↓^*^, T3↓^*^	Fp1↓^*^, Fz↓^*^, T3↓^*^	Pz↑^*^	FP2↓^*^, F3↓^*^, P4↓^*^, F8↓^*^,Fz↓^*^	F4↓^*^, Fz↓^*^
BC	Fz↑^*^, T3↑^*^, O1↑^*^, O2↑^*^	P4↑^*^, Fz↑^*^	C3↑^*^, C4↑^*^, T6↓^*^, Fz↑^*^	C3↓^*^, P3↑^*^, T3↓^*^, T4↓^*^, T5↓^*^, Fz↓^*^, Pz↑^*^	F3↑^*^, F8↑^*^

*Two-sided non-parametric Wilcoxon rank-sum tests were taken*.

* *Indicates statistical significance at P < 0.05*;

*↑ represents increasing or expanding, and*

*↓ represents decreasing or shortening*.

*BC, betweenness centrality; CC, clustering coefficient; CPL, characteristic path length; ND, node degree. SNS, sleep period; WNS, awake period*.

### ND Differences in Varied Frequency Bands

The total period of attacks occurring during the sleep period was too short to meet the EEG requirements; hence, only attacks occurring during the waking period were studied. A comparison of the ictal with interictal EEGs showed larger NDs of the delta band in all channels and that of the theta band in the front of the head (FP2, C3, and C4) (*P* < 0.05). However, the alpha band, principally at the front of the head, (FP1, FP2, C3, Cz, F7, F8, etc.), the beta band in all channels, and the gamma band of the lobes, mainly near the paramedian line and subsylvian region, (FP2, F4, F8, T3, T4, T5, etc.) were smaller (*P* < 0.05) (Figure 3, Table 2).

A comparison of the waking and sleep phases (interictal) showed smaller delta frequencies of the posterior of the head (P3, O1, and O2) (*P* < 0.05) and theta band at the front of the head (FP1, FP2, F4, and C3) (*P* < 0.05). We found a larger alpha band at the front of the head (FP1 and C4), a smaller alpha band of T6, smaller beta bands of the frontal, temporal, and occipital lobes (FP2, F4, F7, F8, T4, T6, O1, and O2), especially in the right hemisphere, a smaller gamma band of Fz, and a larger gamma band of C4 (*P* < 0.05) (Figure 3, Table 3).

### CC Differences Between the Groups

A comparison of the ictal and interictal EEGs showed that the delta frequency band of CC in all channels and the theta band of the temporal and midline regions (T4, T6, Fz, and Cz) expanded. Conversely, the alpha frequency band of the anterior of the head (F4 and Pz) and the beta frequency band in all channels decreased (*P* < 0.05). The gamma band of the paramedian line and left temporal region (FP1, FP2, C4, P4, T3, T5, etc.) declined (*P* < 0.05) (Figure 4, Table 2).

Comparing the waking phases and sleep phases, the alpha band of Pz expanded, but declines were observed for the delta band of CC in the anterior of the head (FP2, Fz, Cz, etc.), the theta band in FP1, Fz, and T3, the beta band of the front of the head (FP2, F3, P4, F8, and Fz), and the gamma band of F4 and Fz (*P* < 0.05) (Figure 4, Table 3).

### BC Differences Between the Groups

A comparison of BC values in the ictal and interictal stages showed that the delta bands of the front of the head (FP1, FP2, F3, F4, C4, F7, F8, etc.) and the theta band of T4 declined, while the alpha band of the front of the head (FP1, F4, and P4) expanded (*P* < 0.05). The beta band of the front of the head and the left occipital lobe (C3, P3, Pz, and O1), the gamma band of the front of the head (FP2, P4, Pz, and T4) expanded (*P* < 0.05) (Figure 5, Table 2).

A comparison of the waking and sleep phases showed that the delta band of the front of the head, in addition to the occipital lobes, (Fz, T3, O1, and O2) and the theta band of the front of the head (P4 and Fz) expanded. The alpha band of the front of the head (C3, C4, and Fz) expanded while that at T6 declined. The beta bands of C3, T3, T4, T5, and Fz declined, while that of the parietal lobe (P3 and Pz) expanded. The gamma band of F3 and F8 expanded. All of the above comparisons yielded significant differences (*P* < 0.05) (Figure 5, Table 3). More details on the original data are available in the Supplementary Materials at the end of the article.

## Discussion

In this study, we conducted the clinical data and used EEG brain functional connectivity analysis to examine the CPL, ND, CC, and BC of 8 IS patients. We discovered that the age of onset of the 8 IS children was 4.42–7.34 months, while the age at the start of treatment was 6.50 (5.25–8.00) months. All 8 patients had psychomotor retardation. These were the same patients as in the previous research on IS (1, 21). The EEGs of 3 patients showed hypsarrhythmia, while the other 5 patients' EEGs showed atypical hypsarrhythmia. We could not easily distinguish or determine the signatures from hypsarrhythmia scalp EEGs of the IS patients. Similarly to a previous study, 62.50% of IS patients' etiology was structural (22). We demonstrated that there were significant differences in functional brain networks between the ictal with interictal onsets and the sleep-wake phase. The changes in CPL, ND, CC, and BC of difference frequency bands correlated with the occurrence of ES (23). CPL, ND, and CC of the fast waves decreased while BC increased. CPL and BC of the slow waves decreased, while ND and CC increased during the IS ictal onset. This was consistent with the prior studies, indicating that patients exhibiting epileptic spasms had stronger functional connections (24). In patients with IS, the overnight reduction in the slope of slow waves was significantly diminished compared to controls (23). Although prior studies have reported that it is difficult to distinguish sleep-wake cycles in IS (25), our report suggests that the CPL of the alpha bands was decreased, and BC was increased during the waking time.

The fast-wave overconnectivity and the slow-wave high local connectivity and defense capabilities may reflect IS pathogenesis, and the alpha band played the most important role during the waking periods, especially in the frontal lobes. Up to 30% of patients with focal epilepsy have seizures originating from the frontal lobe, accounting for the most common extratemporal type (26). We observed that EEG functional network changed when spasms occurred, especially in the forehead, which was different from the brain lesion location on MRI (Figure 1). Researchers have provided important insight into the pathophysiology of ES by performing invasive recording studies that have shown that ES originates locally in the cortex and has been characterized by rapid propagation to the premotor and motor cortices (27). Given the young age of the studied cases, thin skulls are not suitable for invasive recording, but maybe the above research can explain our findings on the characteristics of ictal onset. However, the reason for the active functional network in IS patients' forehead requires further research.

Currently, IS is classified as including a “focal seizure, generalized seizure, or unknown reason seizure” by the ILAE (28). Spasms may involve seizures with focal electrographic onset regardless of visual symmetry (14). The ictal EEG of ES is often characterized by a diffuse burst of slow waves, while low-voltage fast activity superimposes, precedes, or follows behind and is often more focal (29). Combined with interictal focal anomalies, this pattern can help define the epileptogenic zones. Slow waves coupled with high-frequency oscillations can be explained as near-field and locally synchronized potentials generated by the neocortex (14, 29, 30). Furthermore, ES can be associated with a focal leading spike that precedes a fast-wave burst in widespread brain regions (31, 32). Scalp EEG gamma and beta activities may indicate localized seizure onset in IS (14). Our studies showed that the connectivity and defense capabilities of the slow waves increased, and fast waves played more important roles in the global brain network, centrality in the ictal IS region (Table 2, Figures 2–5). The different roles of slow and fast waves in IS EEG network facilitated ES onset.

The sensorimotor cortex is important for ES initiation, while the ripple activity loop between the sensorimotor cortex and resected zone gives rise to seizure onset when talking about patients who have undergone epilepsy surgery (33). In our study, the infant subjects had shorter CPL values in the delta, alpha, and beta bands, suggesting that they played roles in the global network, which was different from the research mentioned above. It was hypothesized that it might be relevant to different EEG monitoring methods and attenuation of fast wave signals through the skull. Meanwhile, local EEG connectivity and defense capability of the slow waves increased, but it reduced in fast waves. Conversely, the centrality of slow waves was weaker while fast waves were stronger, especially in the front of the head. Functional networks may be valuable as objective markers of therapeutic responses to ES, which can facilitate the rapid identification of personalized and effective treatment (7).

Sleep has been studied using synchronized events occurring in billions of coupled neurons in thalamocortical systems. Sleep spindles likely reflect the malleability and strength of thalamocortical circuits (15, 34). Slow-wave sleep downscaling is impaired in IS patients (23). An EEG from a subject with IS shows hypsarrhythmia, while sleep patterns are not easily distinguished from waking patterns. Our findings indicate that the alpha band is stronger in the global and local networks, while slow waves and the beta band are weaker in local connectivity and defense capabilities during waking periods. This suggests that fast waves still play an important role in the waking period of IS, but the dysfunction of slow and fast waves in local connectivity, defense capabilities, and global network leads to difficulty in distinguishing the waking and sleep phases. This has not been reported before and could not be easily discovered by the BASED score. Moreover, although gamma rhythms are commonly observed in many brain regions in waking and sleep states, their mechanisms and functions remain unclear (35). Our comparison of CC values between the waking and sleep phases showed that the gamma band of F4 and Fz declined while BC of F3 and F8 expanded. This is worthy of further study to investigate the role of the gamma band in IS. Our results support the hypothesis that the alpha band in IS plays the most important role during waking periods, but different conditions are applied during sleep.

This study has several limitations. Firstly, we investigated and tried to summarize the common features of different IS etiologies; therefore, the patient cohort contained different types of cases. Secondly, there was no control group in our study in view that there would be no seizures to record. For the sleep-wake phase, we discovered differences in the frequency bands in the EEG network compared with the cranial hypsarrhythmia EEG features, not shown among the IS and control groups. Lastly, we did research on the primitive network features prior to glucocorticoid treatment; hence, post-glucocorticoid results are lacking. We plan to identify more patients with IS due to different etiologies and recruit a control group to determine biomarkers before and after glucocorticoid treatment. This will help clarify the characteristic changes and mechanism of IS, as well as guide the clinical diagnosis and treatment.

## Conclusion

Our findings suggest that fast waves play more important roles in the global brain network and the centrality of the local network, while the local connectivity and defense capabilities of the slow waves are enhanced with IS onset, especially in the front of the head. These may be the signatures IS brain network. Collectively, these results provide different insights into the acknowledgment of IS onset, which will be essential for guiding further brain functional network studies on IS.

## Data Availability Statement

The original contributions presented in the study are included in the article/Supplementary Materials, further inquiries can be directed to the corresponding author.

## Ethics Statement

The studies involving human participants were reviewed and approved by the ethical standards of the Third Affiliated Hospital of Zhengzhou University's Institutional Research Committee (Zhengzhou, China; no. 2021-042-01). Written informed consent from the participants' legal guardian/next of kin was not required to participate in this study in accordance with the national legislation and the institutional requirements.

## Author Contributions

YD made substantial contributions to the study conception and design and agreed to be accountable for all aspects of the work in ensuring that questions related to the accuracy or integrity of any part of the work were appropriately investigated and resolved. RX participated in drafting the manuscript and in acquiring, collecting, analyzing, and interpreting the data. YZ participated in revising the manuscript and in obtaining funding. YS and FW participated in collecting and interpreting the data. KD, TJ, and JW participated in the experimental design and confirmed the authenticity of the raw data. All authors read and approved the final manuscript.

## Funding

Funding was received from the Key Project of Medical Science and Technology of Henan Province (LHGJ20190339), Open Project of the Children's Neural Development Engineering Research Center of Henan Province (SG201910), and Open Research of Henan Key Laboratory of Child Brain Injury and Henan Pediatric Clinical Research Center (KFKT2021003).

## Conflict of Interest

The authors declare that the research was conducted in the absence of any commercial or financial relationships that could be construed as a potential conflict of interest.

## Publisher's Note

All claims expressed in this article are solely those of the authors and do not necessarily represent those of their affiliated organizations, or those of the publisher, the editors and the reviewers. Any product that may be evaluated in this article, or claim that may be made by its manufacturer, is not guaranteed or endorsed by the publisher.
