# Corrigendum: Different frequency bands in various regions of the brain play different roles in the onset and wake-sleep stages of infantile spasms

**DOI:** 10.3389/fped.2023.1142196

**Published:** 2023-02-07

**Authors:** Yan Dong, Ruijuan Xu, Yaodong Zhang, Yali Shi, Kaixian Du, Tianming Jia, Jun Wang, Fang Wang

**Affiliations:** ^1^ Henan Key Laboratory of Child Brain Injury and Henan Pediatric Clinical Research Center, The Third Affiliated Hospital and Institute of Neuroscience, Zhengzhou, China; ^2^ Henan Key Laboratory of Children’s Genetics and Metabolic Diseases, Henan Neurodevelopment Engineering Research Center for Children, Children’s Hospital Affiliated to Zhengzhou University, Zhengzhou, China; ^3^ Department of Children’s Rehabilitation, The Third Affiliated Hospital of Zhengzhou University, Zhengzhou, China; ^4^ Department of Medical Record Management, The Third Affiliated Hospital of Zhengzhou University, Zhengzhou, China

**Keywords:** EEG analysis, infantile spasm, functional brain networks, local network, global network

Corrigendum on Different frequency bands in various regions of the brain play different roles in the onset and wake-sleep stages of infantile spasms Dong Y, Xu R, Zhang Y, Shi Y, Du K, Jia T, et al. (2022) Front. Pediatr. 10:878099. doi: 10.3389/fped.2022.878099

In the published article, there was an error in institution namesof affiliations 1, 3 and 4. The original affiliations “^1^Henan Provincial Key Laboratory of Child Brain Injury, Department of Pediatrics, Third Associated Hospital of ZhengZhou University, Zhengzhou, China”, “^3^Department of Children’s Rehabilitation, Third Associated Hospital of ZhengZhou University, Zhengzhou, China” and “^4^Department of Medical Record Management, Third Associated Hospital of ZhengZhou University, Zhengzhou, China”, have been changed to “^1^Henan Key Laboratory of Child Brain Injury and Henan Pediatric Clinical Research Center, the Third Affiliated Hospital and Institute of Neuroscience, Zhengzhou, China”, “^3^Department of Children's Rehabilitation, The Third Affiliated Hospital of Zhengzhou University, Zhengzhou, China” and “^4^Department of Medical Record Management, The Third Affiliated Hospital of Zhengzhou University, Zhengzhou, China”.

The authors apologize for this error and state that this does not change the scientific conclusions of the article in any way. The original article has been updated.

