# Supplementary material for: Different Frequency Bands in Various Regions of the Brain Play Different Roles in the Onset and Wake-Sleep Stages of Infantile Spasms
Source: Front Pediatr. 2022 May 12;10:878099. doi: 10.3389/fped.2022.878099 (PMC9135356; doi:10.3389/fped.2022.878099)
Supplement: Supplementary file 1 [file Data_Sheet_1.PDF]

1. CPL in different frequency bands of IS compared WS with WNS stage.

| 频段 | *Z* | *P* |
|---|---|---|
| Delta | -8.891 | 6.0790E-19 |
| Theta | -0.271 | 0.7865 |
| Alpha | -2.028 | 0.0426 |
| Beta | -5.598 | 2.1722E-08 |
| Gamma | -0.444 | 0.6571 |

2. ND in different frequency bands of IS compared WS with WNS stage.

| 节点 | ND delta | | ND theta | | ND alpha | | ND beta | | ND gamma | |
|---|---|---|---|---|---|---|---|---|---|---|
| | *Z* | *P* | *Z* | *P* | *Z* | *P* | *Z* | *P* | *Z* | *P* |
| Fp1 | -9.404 | 0 | -0.418 | 0.676 | -2.73 | 0.006 | -8.517 | 0 | -1.095 | 0.273 |
| Fp2 | -8.196 | 0 | -2.343 | 0.019 | -3.026 | 0.002 | -8.655 | 0 | -5.03 | 0 |
| F3 | -8.542 | 0 | -0.79 | 0.43 | 0 | 1 | -6.956 | 0 | -1.056 | 0.291 |
| F4 | -7.515 | 0 | -0.75 | 0.453 | -1.159 | 0.246 | -7.342 | 0 | -1.985 | 0.047 |
| C3 | -9.119 | 0 | -2.058 | 0.04 | -2.043 | 0.041 | -6.288 | 0 | -1.474 | 0.141 |
| C4 | -7.384 | 0 | -1.967 | 0.049 | -1.339 | 0.181 | -6.745 | 0 | -1.127 | 0.26 |
| P3 | -9.205 | 0 | -0.805 | 0.421 | -0.693 | 0.489 | -5.331 | 0 | -1.465 | 0.143 |
| P4 | -9.782 | 0 | -1.243 | 0.214 | -0.425 | 0.671 | -6.457 | 0 | -0.663 | 0.508 |
| O1 | -9.406 | 0 | -0.183 | 0.855 | -1.227 | 0.22 | -4.554 | 0 | -1.883 | 0.06 |
| O2 | -8.889 | 0 | -1.551 | 0.121 | -2.229 | 0.026 | -6.578 | 0 | -2.376 | 0.018 |
| F7 | -8.342 | 0 | -1.842 | 0.066 | -2.23 | 0.026 | -7.492 | 0 | -1.406 | 0.16 |
| F8 | -6.027 | 0 | -1.836 | 0.066 | -2.674 | 0.007 | -7.045 | 0 | -2.135 | 0.033 |
| T3 | -4.972 | 0 | -1.041 | 0.298 | -1.394 | 0.163 | -7.553 | 0 | -2.354 | 0.019 |
| T4 | -7.262 | 0 | -0.389 | 0.697 | -0.784 | 0.433 | -6.046 | 0 | -2.146 | 0.032 |
| T5 | -8.706 | 0 | -1.757 | 0.079 | -1.026 | 0.305 | -8.24 | 0 | -2.928 | 0.003 |
| T6 | -8.502 | 0 | -1.587 | 0.113 | -0.532 | 0.595 | -6.159 | 0 | -1.576 | 0.115 |
| Fz | -7.687 | 0 | -0.433 | 0.665 | -1.953 | 0.051 | -6.868 | 0 | -0.223 | 0.823 |
| Cz | -7.995 | 0 | -0.667 | 0.505 | -2.846 | 0.004 | -5.557 | 0 | -0.622 | 0.534 |
| Pz | -8.596 | 0 | -1.44 | 0.15 | -0.435 | 0.663 | -5.456 | 0 | -0.818 | 0.413 |

3. CC in different frequency bands of IS compared WS with WNS stage.

| 节点 | CC delta | | CC theta | | CC alpha | | CC beta | | CC gamma | |
|---|---|---|---|---|---|---|---|---|---|---|
| | *Z* | *P* | *Z* | *P* | *Z* | *P* | *Z* | *P* | *Z* | *P* |
| Fp1 | -7.604 | 0 | -1.47 | 0.142 | -0.848 | 0.396 | -5.531 | 0 | -2.389 | 0.017 |
| Fp2 | -9.217 | 0 | -1.169 | 0.242 | -0.585 | 0.558 | -7.102 | 0 | -2.346 | 0.019 |
| F3 | -8.828 | 0 | -1.505 | 0.132 | -0.686 | 0.493 | -5.294 | 0 | -1.373 | 0.17 |
| F4 | -7.997 | 0 | -1.246 | 0.213 | -2.477 | 0.013 | -6.409 | 0 | -1.084 | 0.278 |
| C3 | -9.374 | 0 | -1.377 | 0.169 | -0.908 | 0.364 | -7.242 | 0 | -1.494 | 0.135 |
| C4 | -9.93 | 0 | -1.498 | 0.134 | -0.779 | 0.436 | -6.872 | 0 | -2.204 | 0.028 |
| P3 | -8.453 | 0 | -0.588 | 0.557 | -0.922 | 0.356 | -6.347 | 0 | -1.849 | 0.064 |
| P4 | -7.097 | 0 | -0.911 | 0.363 | -1.618 | 0.106 | -6.661 | 0 | -3.367 | 0.001 |
| O1 | -8.636 | 0 | -1.624 | 0.104 | -1.322 | 0.186 | -5.662 | 0 | -0.975 | 0.33 |
| O2 | -7.735 | 0 | -0.763 | 0.445 | -0.456 | 0.649 | -6.08 | 0 | -1.966 | 0.049 |
| F7 | -9.05 | 0 | -1.455 | 0.146 | -1.408 | 0.159 | -6.639 | 0 | -1.086 | 0.277 |
| F8 | -7.707 | 0 | -0.152 | 0.879 | -0.61 | 0.542 | -4.787 | 0 | -1.453 | 0.146 |
| T3 | -10.628 | 0 | -1.687 | 0.092 | -1.238 | 0.216 | -7.564 | 0 | -3.141 | 0.002 |
| T4 | -9.113 | 0 | -3.37 | 0.001 | -0.386 | 0.699 | -4.895 | 0 | -1.18 | 0.238 |
| T5 | -6.904 | 0 | -0.244 | 0.807 | -1.077 | 0.281 | -7.264 | 0 | -3.149 | 0.002 |
| T6 | -10.476 | 0 | -3.078 | 0.002 | -1.083 | 0.279 | -4.844 | 0 | -1.064 | 0.287 |
| Fz | -9.989 | 0 | -2.361 | 0.018 | -1.077 | 0.282 | -6.25 | 0 | -0.924 | 0.355 |
| Cz | -11.033 | 0 | -1.976 | 0.048 | -0.988 | 0.323 | -6.634 | 0 | -0.059 | 0.953 |
| Pz | -8.891 | 0 | -0.271 | 0.786 | -2.028 | 0.043 | -5.598 | 0 | -0.444 | 0.657 |

4. BC in different frequency bands of IS compared WS with WNS stage.

| 节点 | BC delta | | BC theta | | BC alpha | | BC beta | | BC gamma | |
|---|---|---|---|---|---|---|---|---|---|---|
| | *Z* | *P* | *Z* | *P* | *Z* | *P* | *Z* | *P* | *Z* | *P* |
| Fp1 | -2.526 | 0.012 | -1.712 | 0.087 | -2.242 | 0.025 | -0.316 | 0.752 | -1.005 | 0.315 |
| Fp2 | -2.676 | 0.007 | -0.633 | 0.527 | -1.489 | 0.136 | -0.37 | 0.712 | -2.053 | 0.04 |
| F3 | -2.094 | 0.036 | -1.004 | 0.315 | -1.66 | 0.097 | -0.399 | 0.69 | -0.512 | 0.609 |
| F4 | -3.494 | 0 | -1.073 | 0.283 | -2.177 | 0.029 | -1.007 | 0.314 | -0.244 | 0.807 |
| C3 | -1.052 | 0.293 | -0.268 | 0.789 | -1.192 | 0.233 | -2.7 | 0.007 | -0.476 | 0.634 |
| C4 | -3.562 | 0 | -0.419 | 0.675 | -0.258 | 0.796 | -0.314 | 0.754 | -0.272 | 0.785 |
| P3 | -0.621 | 0.534 | -1.101 | 0.271 | -1.389 | 0.165 | -2.375 | 0.018 | -0.365 | 0.715 |
| P4 | -0.021 | 0.983 | -0.885 | 0.376 | -3.059 | 0.002 | -1.274 | 0.203 | -2.047 | 0.041 |
| O1 | -0.231 | 0.817 | -1.147 | 0.251 | -0.08 | 0.936 | -3.936 | 0 | -0.927 | 0.354 |
| O2 | -0.075 | 0.94 | -0.883 | 0.377 | -0.555 | 0.579 | -0.655 | 0.512 | -0.161 | 0.872 |
| F7 | -2.229 | 0.026 | -0.931 | 0.352 | -0.419 | 0.675 | -0.453 | 0.651 | -0.76 | 0.447 |
| F8 | -3.384 | 0.001 | -1.431 | 0.152 | -1.24 | 0.215 | -0.16 | 0.873 | -0.553 | 0.58 |
| T3 | -4.584 | 0 | -0.473 | 0.636 | -0.073 | 0.942 | -1.106 | 0.269 | -1.056 | 0.291 |
| T4 | -2.887 | 0.004 | -3.397 | 0.001 | -0.187 | 0.852 | -0.595 | 0.552 | -2.177 | 0.029 |
| T5 | -1.856 | 0.064 | -0.338 | 0.735 | -0.028 | 0.978 | -0.238 | 0.812 | -0.709 | 0.478 |
| T6 | -3.213 | 0.001 | -1.563 | 0.118 | -1.822 | 0.069 | -0.016 | 0.987 | -0.336 | 0.737 |
| Fz | -4.76 | 0 | -1.246 | 0.213 | -1.649 | 0.099 | -0.091 | 0.928 | -0.97 | 0.332 |
| Cz | -3.214 | 0.001 | -0.287 | 0.774 | -1.18 | 0.238 | -1.119 | 0.263 | -1.205 | 0.228 |
| Pz | -1.717 | 0.086 | -0.165 | 0.869 | -1.294 | 0.196 | -2.032 | 0.042 | -2.232 | 0.026 |

、

5. CPL in different frequency bands of IS between the WNS and SNS stages

| 频段 | *Z* | *P* |
|---|---|---|
| Delta | -1.572 | 0.1161 |
| Theta | -0.582 | 0.5610 |
| Alpha | -2.172 | 0.0299 |
| Beta | -1.143 | 0.2531 |
| Gamma | -0.311 | 0.7558 |

6. ND in different frequency bands of IS between the WNS and SNS stages.

| 节点 | ND delta | | ND theta | | ND alpha | | ND beta | | ND gamma | |
|---|---|---|---|---|---|---|---|---|---|---|
| | *Z* | *P* | *Z* | *P* | *Z* | *P* | *Z* | *P* | *Z* | *P* |
| Fp1 | -0.874 | 0.382 | -2.323 | 0.02 | -2.203 | 0.028 | -1.783 | 0.075 | -0.364 | 0.716 |
| Fp2 | -1.056 | 0.291 | -2.673 | 0.008 | -0.456 | 0.649 | -3.851 | 0 | -1.508 | 0.132 |
| F3 | -0.183 | 0.855 | -1.824 | 0.068 | -0.109 | 0.913 | -0.96 | 0.337 | -0.706 | 0.48 |
| F4 | -0.32 | 0.749 | -2.338 | 0.019 | -0.668 | 0.504 | -3.567 | 0 | -1.281 | 0.2 |
| C3 | -0.857 | 0.392 | -2.257 | 0.024 | -1.664 | 0.096 | -0.226 | 0.821 | -1.792 | 0.073 |
| C4 | -1.673 | 0.094 | -1.542 | 0.123 | -2.007 | 0.045 | -1.349 | 0.177 | -2.309 | 0.021 |
| P3 | -2.343 | 0.019 | -1.113 | 0.266 | -1.195 | 0.232 | -0.653 | 0.514 | -0.793 | 0.428 |
| P4 | -1.184 | 0.237 | -0.518 | 0.605 | -1.169 | 0.243 | -1.272 | 0.203 | -0.463 | 0.643 |
| O1 | -3.42 | 0.001 | -1.367 | 0.172 | -1.243 | 0.214 | -2.187 | 0.029 | -0.858 | 0.391 |
| O2 | -3.128 | 0.002 | -1.69 | 0.091 | -0.021 | 0.983 | -2.352 | 0.019 | -0.818 | 0.413 |
| F7 | -0.213 | 0.831 | -1.323 | 0.186 | -0.046 | 0.963 | -2.874 | 0.004 | -0.966 | 0.334 |
| F8 | -0.113 | 0.91 | -1.19 | 0.234 | -1.794 | 0.073 | -3.023 | 0.003 | -0.097 | 0.923 |
| T3 | -1.176 | 0.24 | -1.094 | 0.274 | -0.479 | 0.632 | -0.645 | 0.519 | -1.065 | 0.287 |
| T4 | -0.069 | 0.945 | -0.476 | 0.634 | -1.177 | 0.239 | -3.859 | 0 | -1.221 | 0.222 |
| T5 | -1.919 | 0.055 | -1.65 | 0.099 | -1.218 | 0.223 | -0.136 | 0.892 | -0.798 | 0.425 |
| T6 | -0.141 | 0.888 | -1.321 | 0.187 | -2.172 | 0.03 | -1.997 | 0.046 | -1.021 | 0.307 |
| Fz | -0.209 | 0.835 | -0.83 | 0.406 | -1.807 | 0.071 | -1.594 | 0.111 | -2.48 | 0.013 |
| Cz | -0.799 | 0.424 | -0.758 | 0.448 | -1.013 | 0.311 | -1.426 | 0.154 | -1.71 | 0.087 |
| Pz | -0.071 | 0.944 | -1.443 | 0.149 | -1.064 | 0.287 | -1.565 | 0.118 | -0.284 | 0.777 |

7. CC in different frequency bands of IS between the WNS and SNS stages.

| 节点 | CC delta | | CC theta | | CC alpha | | CC beta | | CC gamma | |
|---|---|---|---|---|---|---|---|---|---|---|
| | *Z* | *P* | *Z* | *P* | *Z* | *P* | *Z* | *P* | *Z* | *P* |
| Fp1 | -0.244 | 0.808 | -2.109 | 0.035 | -0.017 | 0.986 | -1.102 | 0.271 | -0.787 | 0.431 |
| Fp2 | -2.549 | 0.011 | -0.596 | 0.551 | -1.491 | 0.136 | -2.862 | 0.004 | -0.275 | 0.784 |
| F3 | -0.041 | 0.967 | -1.861 | 0.063 | -0.016 | 0.988 | -2.222 | 0.026 | -0.035 | 0.972 |
| F4 | -0.25 | 0.803 | -1.044 | 0.296 | -1.203 | 0.229 | -1.508 | 0.132 | -2.029 | 0.042 |
| C3 | -1.482 | 0.138 | -1.231 | 0.218 | -0.045 | 0.964 | -0.266 | 0.79 | -0.223 | 0.824 |
| C4 | -0.535 | 0.593 | -1.134 | 0.257 | -0.388 | 0.698 | -0.106 | 0.916 | -1.155 | 0.248 |
| P3 | -0.578 | 0.564 | -0.757 | 0.449 | -0.095 | 0.924 | -0.519 | 0.604 | -0.146 | 0.884 |
| P4 | -0.456 | 0.648 | -0.89 | 0.373 | -0.589 | 0.556 | -2.109 | 0.035 | -0.651 | 0.515 |
| O1 | -1.486 | 0.137 | -0.747 | 0.455 | -0.478 | 0.633 | -1.489 | 0.136 | -0.484 | 0.628 |
| O2 | -0.462 | 0.644 | -1.513 | 0.13 | -0.554 | 0.58 | -1.591 | 0.112 | -1.153 | 0.249 |
| F7 | -0.497 | 0.619 | -0.228 | 0.82 | -0.63 | 0.529 | -1.528 | 0.127 | -0.842 | 0.4 |
| F8 | -0.575 | 0.565 | -0.009 | 0.992 | -0.471 | 0.638 | -3.209 | 0.001 | -0.962 | 0.336 |
| T3 | -3.544 | 0 | -2.568 | 0.01 | -0.665 | 0.506 | -1.723 | 0.085 | -1.474 | 0.14 |
| T4 | -0.08 | 0.936 | -1.068 | 0.285 | -0.052 | 0.959 | -1.475 | 0.14 | -1.661 | 0.097 |
| T5 | -1.38 | 0.167 | -0.873 | 0.383 | -0.277 | 0.781 | -1.756 | 0.079 | -0.613 | 0.54 |
| T6 | -1.765 | 0.078 | -1.86 | 0.063 | -1.289 | 0.198 | -1.31 | 0.19 | -0.14 | 0.889 |
| Fz | -3.892 | 0 | -3.684 | 0 | -1.344 | 0.179 | -3.215 | 0.001 | -2.944 | 0.003 |
| Cz | -2.625 | 0.009 | -0.461 | 0.645 | -0.256 | 0.798 | -1.383 | 0.167 | -0.814 | 0.416 |
| Pz | -1.572 | 0.116 | -0.582 | 0.561 | -2.172 | 0.03 | -1.143 | 0.253 | -0.311 | 0.756 |

8. BC in different frequency bands of IS between the WNS and SNS stages.

| 节点 | BC delta | | BC theta | | BC alpha | | BC beta | | BC gamma | |
|---|---|---|---|---|---|---|---|---|---|---|
| | *Z* | *P* | *Z* | *P* | *Z* | *P* | *Z* | *P* | *Z* | *P* |
| Fp1 | -0.062 | 0.95 | -0.001 | 0.999 | -1.467 | 0.142 | -1.051 | 0.293 | -0.751 | 0.452 |
| Fp2 | -1.016 | 0.31 | -1.124 | 0.261 | -1.465 | 0.143 | -1.573 | 0.116 | -0.859 | 0.39 |
| F3 | -1.332 | 0.183 | -0.381 | 0.703 | -0.104 | 0.917 | -1.651 | 0.099 | -1.995 | 0.046 |
| F4 | -0.98 | 0.327 | -0.437 | 0.662 | -0.641 | 0.521 | -0.571 | 0.568 | -0.371 | 0.71 |
| C3 | -0.692 | 0.489 | -0.189 | 0.85 | -2.279 | 0.023 | -2.169 | 0.03 | -1.811 | 0.07 |
| C4 | -0.538 | 0.591 | -1.177 | 0.239 | -2.399 | 0.016 | -1.791 | 0.073 | -1.751 | 0.08 |
| P3 | -1.156 | 0.248 | -0.351 | 0.726 | -1.107 | 0.268 | -2.794 | 0.005 | -0.429 | 0.668 |
| P4 | -1.43 | 0.153 | -2.027 | 0.043 | -1.598 | 0.11 | -0.902 | 0.367 | -0.018 | 0.985 |
| O1 | -3.072 | 0.002 | -0.13 | 0.896 | -0.51 | 0.61 | -0.309 | 0.758 | -0.337 | 0.736 |
| O2 | -3.13 | 0.002 | -0.839 | 0.401 | -0.153 | 0.878 | -1.384 | 0.166 | -0.069 | 0.945 |
| F7 | -1.256 | 0.209 | -0.34 | 0.734 | -1.178 | 0.239 | -1.457 | 0.145 | -1.227 | 0.22 |
| F8 | -0.875 | 0.381 | -0.451 | 0.652 | -1.597 | 0.11 | -0.245 | 0.806 | -2.103 | 0.035 |

| | | | | | | | | | | |
|---|---|---|---|---|---|---|---|---|---|---|
| T3 | -3.284 | 0.001 | -1.924 | 0.054 | -1.67 | 0.095 | -2.444 | 0.015 | -0.291 | 0.771 |
| T4 | -0.539 | 0.59 | -1.898 | 0.058 | -1.226 | 0.22 | -2.135 | 0.033 | -0.425 | 0.671 |
| T5 | -0.712 | 0.476 | -1.114 | 0.265 | -0.373 | 0.709 | -2.41 | 0.016 | -1.07 | 0.284 |
| T6 | -0.25 | 0.803 | -1.468 | 0.142 | -2.561 | 0.01 | -0.287 | 0.774 | -0.845 | 0.398 |
| Fz | -3.486 | 0 | -2.166 | 0.03 | -4.023 | 0 | -2.156 | 0.031 | -0.281 | 0.779 |
| Cz | -1.465 | 0.143 | -0.467 | 0.64 | -0.562 | 0.574 | -0.846 | 0.397 | -1.526 | 0.127 |
| Pz | -1.294 | 0.196 | -0.455 | 0.649 | -1.577 | 0.115 | -4.242 | 0 | -0.233 | 0.816 |

All red color showed $P \leq 0.05$
